# Severe testing with high-dimensional omics data for enhancing biomedical scientific discovery

**DOI:** 10.1038/s41540-022-00251-8

**Published:** 2022-10-21

**Authors:** Frank Emmert-Streib

**Affiliations:** Predictive Society and Data Analytics Lab, Faculty of Information Technology and Communication Sciences, Tampere University, Tampere, Finland

**Keywords:** Systems analysis, Biomarkers, Biomarkers, Statistics, Software

## Abstract

High-throughput omics experiments provide a wealth of data for exploring biomedical questions and for advancing translational research. However, despite this great potential, results that enter the clinical practice are scarce even twenty years after the completion of the human genome project. For this reason in this paper, we revisit problems with scientific discovery commonly summarized under the term reproducibility crisis. We will argue that the major problem that hampers progress in translational research is threefold. First, in order to establish biological foundations of disorders or general complex phenotypes, one needs to embrace emergence. Second, there seems to be confusion about the underlying hypotheses tested by omics studies. Third, most contemporary omics studies are designed to perform what can be seen as incremental corroborations of a hypothesis. In order to improve upon these shortcomings, we define a severe testing framework (STF) that can be applied to a large number of omics studies for enhancing scientific discovery in the biomedical sciences. Briefly, STF provides systematic means to trim wild-grown omics studies in a constructive way.

## Introduction

During the last almost three decades, we have witnessed unprecedented progress in biology and the biomedical sciences^1,2^. Triggered by technological advances of high-throughput technologies and computing power, the analysis of big omics data challenged our established method of scientific discovery. Specifically, hypothesis-driven research, predominating the physical sciences, seems to have been replaced by induction-based research. Some pioneers of high-throughput technologies even stated, "the patterns of expression will often suffice to begin de novo discovery of potential gene functions”^3^.

Looking back, it is undeniable that this past time period has been very productive and one milestone thereof is the human genome project^4^. However, it is also indisputable that there are major problems that cast shadows on the initial euphoria, especially in the context of translational research. In recent years, an antagonist of the latter has been called the replication crisis^5,6^. But even before this, general concerns have been raised against about omics studies with arguments centered around genetic determinism^7^.

In this paper, we want to take a fundamental look at these problems. That means instead of discussing problems within the existing framework of omics studies, e.g., by addressing particular issues with statistical methodologies, animal models, or data quality^8^, we approach these via the method of scientific discovery. Due to the fact that scientific discovery is usually largely omitted from such considerations, we start with discussing major methods thereof which will provide us with insights about limitations and opportunities. Based on this, we will provide a discussion of problems in general omics studies with complex phenotypes. We will see that most contemporary genomics studies are designed to perform what can be seen as incremental corroborations of a hypothesis. Hence, such studies are by design prone to make little advances. In order to improve upon these shortcomings, we define a severe testing framework (STF) that can be applied to a large number of omics studies for enhancing scientific discoveries in the biomedical sciences by exploiting the full potential of high-dimensional data.

## Scientific reasoning

### Base forms of inference

**Fig. 1:**
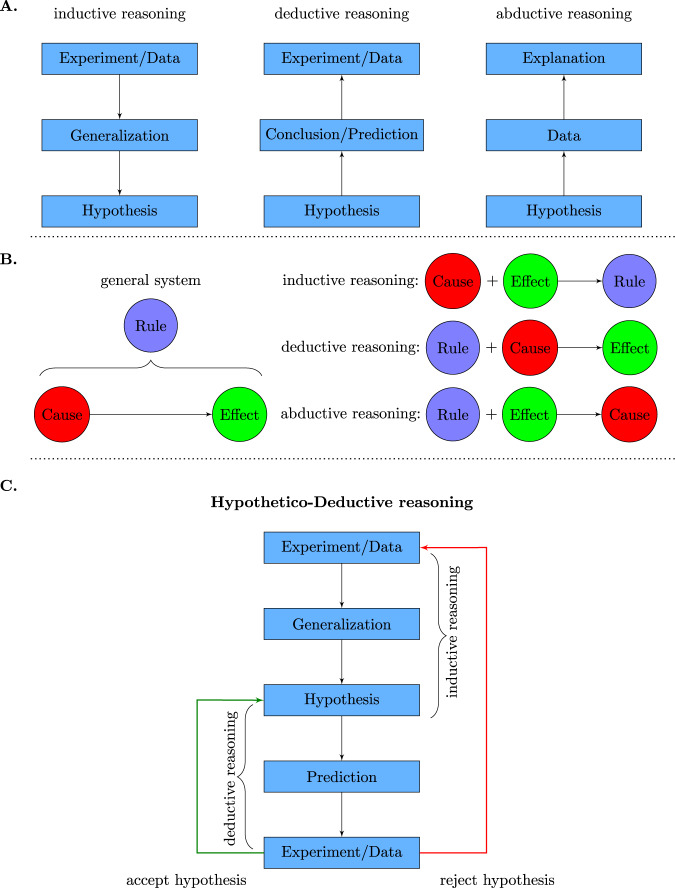
Overview of three different inference approaches and hypothetico-deductive reasoning. **A**, **B** The three base forms of inference: inductive inference, deductive inference, and abductive inference. **C** Basic components and working mechanism of the hypothetico-deductive (HD) method.

There are three main forms of inference or reasoning to distinguish: Induction, deduction, and abduction. In Fig. 1, we show an overview of these three base inference forms. In order to simplify the understanding of their complex meaning, we show two different versions in Fig. 1A and B, respectively. Put simply, inductive reasoning tries to infer from the “special” to the “general”, whereas deductive reasoning tries to infer from the “general” to the “special”. In contrast, abductive inference tries to infer an explanation for a given hypothesis and data. According to ref. ^9^, Peirce describes the differences among the three inferences types as follows: “deduction proves that something must be; induction shows that something actually is operative; abduction merely suggests that something may be”. This implies also that new hypotheses or ideas can only be created by abduction^10^. On a brief historic note, we would like to mention that inductive reasoning goes back to John Stuart Mill, deductive reasoning to Rene Descartes, and abductive reasoning has been introduced by Charles Sanders Peirce. Succinctly, one can summarize the above inference methods as follows. Induction is data-driven, the deduction is theory-driven, and abduction is explanation-driven research^11^.

Importantly, there seems to be no generally accepted meaning of abductive reasoning. As a reason for this, it has been noted that “Peirce went through a substantial change of mind”^12^. Here, we follow^9^ corresponding to the latter view of Peirce on abduction. An important consequence of this confusion is that abductive reasoning has been falsely called “reasoning to the best explanation”^9^. However, inference to the best explanation is supposed to be the last stage of inquiry, whereas abduction corresponds to the first stage of inquiry. Hence, abduction is a method for arriving at hypotheses and selecting a hypothesis to test.

One commonality of all three base forms of inference discussed above is that they can be seen as one-step processes. That means each one has a defined starting and a defined ending point (in Fig. 1A, B, this is indicated by the direction of the arrows), and no iteration over the components occurs in the form of repetition. With respect to the working mechanism of general scientific discovery, this seems inadequate. For this reason, extensions to these base forms of inference have been introduced.

### Hypothetico-deductive method

Maybe the most important extension of the above three base forms of inference is the hypothetico-deductive (HD) method^13^. The HD method has been popularized by Hempel and Popper^14,15^ with early contributions dating back to William Whewell (1794–1866), William Stanley Jevons (1835–1882), and Charles S. Peirce (1838–1914). The basic idea of the HD method is the formulation of a testable hypothesis and its testing^16,17^.

There are variations of the HD method, but its basic components and working mechanism is as follows^18^. (1) Conduct an experiment to generate data, (2) generalize the observations with inductive reasoning by (3) formulating a hypothesis, (4) deduce new predictions from the hypothesis that can be observed, and (5) conduct a new experiment to test if those predictions are true. If they are true, accept the hypothesis and go back to step 3 to deduce new predictions. If they are false, the hypothesis is falsified (reject hypothesis), and one starts again at step 1.

Despite the cyclic nature of the HD method, its side branches, as shown in Fig. 1C, are frequently omitted, resulting in a linear process^19^. While this omission may not be deliberate most of the current science is lacking explicit iterations. This lack of iterations is observable in essentially every paper published. Instead, the iteration is obtained over a series of published papers studying the same underlying problem.

### Further extended models

Aside from the HD method discussed above, there are a number of further extended methods aiming to improve upon the hypthetico-deductive method. Exemplarily, we would like to highlight the cyclic deductive-abductive (CDA) model proposed in ref. ^20^.

The CDA model combines a hypothetico-deductive and an abductive epistemological framework in a cyclic way. That means in the CDA framework, prediction and postdiction cycle continuously, whereas prediction follows the hypothetico-deductive process, and the postdiction is abductive. All exploratory analyses are abductive in nature, and all hypothetico-deductive experiments start from a postdiction, i.e., preliminary evidence suggesting one plausible hypothesis to be tested. By deduction, hypotheses generate new data and findings that, by abduction, refine the hypothesis space for the deduction. Applications and discussions of the CDA method can be found in different domains, e.g., refs. ^21,22^.

Other examples for extended models include hypothetico-inductive inference^23^, strong inference^24^, or allochthonous models^25^. It is important to highlight that regardless of the specific form of a scientific method, each is based on (a subset of) the three base forms of scientific reasoning: induction, deduction, and abduction^26^. The reason for this is that all aspects of inference, i.e., data-driven, theory-driven, and explanation-driven, seem to be needed for corroborating a theory as good as possible with all means available.

### Key elements of scientific discovery: asymmetry, uncertainty, and cyclicity

From the HD method and its extensions for scientific discovery, one can identify three commonalities in addition to the three base forms of inference. These common elements of the models are:

1. Asymmetry
2. Uncertainty
3. Cyclicity

In the following, we will briefly discuss these elements.

The ultimate goal of any scientific method is the verification of a hypothesis. However, to this day, there is no solution known to empirically verify a hypothesis, e.g., by experiments or observations, but only its falsification. This establishes an asymmetry between verification and falsification in the empirical or experimental sciences^27,28^. In turn, this asymmetry is related to the uncertainty of inductive reasoning which does never result in certain knowledge^29^. The third common element of the hypothetico-deductive (HD), hypothetico-deductive and abductive (CDA)^20^, strong inference or other models is that they are applied cyclicly or iteratively^13,25^. The reason for this is related to the uncertainty of inductive and abductive methods. That means a test that does not lead to the falsification of a hypothesis contributes only to its corroboration but not confirmation^15,30^. Hence, by the iterative testing of such methods, the confidence in a hypothesis can be slowly increased over many cycles.

From this discussion, one can see that the above key elements do not provide independent dimensions of scientific discovery but are intricately related to each other.

### Asymptotic reasoning

**Fig. 2:**
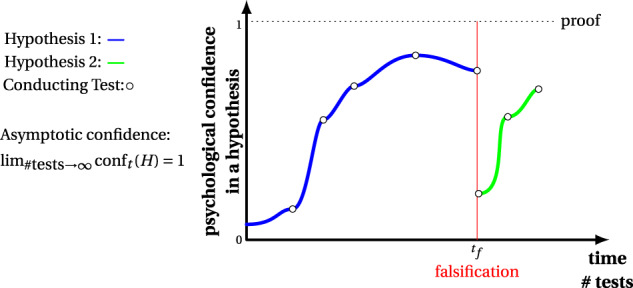
Confidence in a hypothesis over the number of conducted tests (over time). If one of these tests falsifies the hypothesis (blue curve), it needs to be abandoned, and a new hypothesis (green curve) needs to be formulated.

In order to connect this discussion with the problems of omics studies below, the cyclicity of scientific discovery is of special importance. For this reason, we want to take a closer look at some details. In Fig. 2, we depict an example showing the process of corroboration of a hypothesis over time^30^. In the following, we assume a hypothesis is dichotomous, i.e., it is either true or false. In this figure, the two curves (blue and green) corresponding to two different hypotheses, and the *y* axis gives confidence in a hypothesis, *conf*_*t*_(*H*), at time *t*. Each test that does not falsify a hypothesis, potentially contributes to a change in our confidence about the correctness of the hypothesis.

Three points need to be highlighted. First, regardless of what level of confidence in a hypothesis has already been reached, as soon as a test falsifies a hypothesis it needs to be abandoned. An example for this is represented by hypothesis 1 (blue curve) in Fig. 2 which is falsified at time *t*_*f*_. Second, the confidence in a hypothesis reaches only certainty in the asymptotic limit after infinite many tests have been conducted, i.e.,

1 $$\mathop{\lim }\limits_{\#{{{\rm{tests}}}}\to \infty }{{{{\rm{conf}}}}}_{t}(H)=1.$$

This implies it will take an infinite amount of time. In other words, in reality, i.e., where a hypothesis can only be tested for a finite number of times, certainty cannot be reached.

The third point we would like to emphasize is that due to our inability to identify when a hypothesis has been proven, one cannot quantify the confidence, i.e., *conf*_*t*_(*H*) in absolute terms, i.e., objectively. For this reason, *conf*_*t*_(*H*) corresponds to a *psychological* confidence of an individual in hypothesis *H*, which is subjective. This implies that the visualizations in Fig. 2 correspond merely to hypothetical curves providing an exemplification for the effect of tests on the confidence in a hypothesis but another individual may assign different numbers of confidence to the conducted tests. Importantly, the psychological confidence in a hypothesis does not have to be monotonous until a disproof but a test can reduce it, e.g., due to unmet expectations of an outcome. Formally, this could be obtained by choice of different priors when conducting a Bayesian inference^31–33^ and defining “confidence” as the probability of hypothesis H to be true. In the statistics literature, such subjective or epistemic probabilities are well-known giving a subjective status by regarding it as a measure of the “degree of belief” of an individual^34,35^. For completeness, we would like to add that in philosophy, the term *verisimilitude*, meaning closeness to the truth or degree of truthlikeness, has been introduced by Popper^36^ as a means to order different hypotheses with respect to their distance to the truth. However, while its underlying idea is appealing, it has been strongly criticized^37,38^, and to this day no general agreement about its quantification has been reached.

With regard to the structure of the hypothetico-deductive (HD) method, see Fig. 1C, the blue curve in Fig. 2 until the point of falsification reflects only the left part of the HD model. The modification of a hypothesis due to a falsification, corresponding to the right part of the HD model, starts a new process for the corroboration of a new/revised hypothesis. In Fig. 2, this is represented by the green curve corresponding to the new/revised hypothesis 2. This description emphasizes that there are two cycles in a HD method. One is contributing to the corroboration of a hypothesis (blue curve), whereas the other falsifies it and initiates by this a new corroboration for a new/revised hypothesis (green curve).

### Severe testing

There is another topic that connects directly to cyclicity and asymptotic reasoning, and that is the *quality* of a tested hypothesis. In the previous section, we argued that consecutive testing leads to an increase in our confidence in a hypothesis. However, we did not discuss why the step heights of tests, as shown in Fig. 2, are not equal.

The reason for unequal step heights in the corroboration of a hypothesis is related to the quality of a tested hypothesis. Specifically, Popper put great emphasis on the idea of a *severe test* as opposed to tests that involve evidence similar to that already gathered in support of a theory^15,39^. In ref. ^40^, he wrote:

Observations or experiments can be accepted as supporting a theory (or a hypothesis, or a scientific assertion) only if these observations or experiments are severe tests of the theory—or, in other words, only if they result from serious attempts to refute the theory, and especially from trying to find faults where these might be expected in the light of all our knowledge, including our knowledge of competing theories.

It is clear that non-serious attempts to refute a hypothesis can easily lead to a confirmation, however, such a confirmation does not lead to a large increase in the confidence of a hypothesis. Hence, from a scientific perspective, one should always strive to formulate a hypothesis that provides a severe test for the underlying theory.

On a technical note, we would like to mention that Popper did not provide a quantitative formulation of severe testing. Instead, a realization in a statistical hypothesis testing framework has been presented in ref. ^41^.

## Problems with scientific discovery in omics

After this general discussion of different forms of scientific reasoning and its key elements, we now address specific problems with this encountered in contemporary omics studies.

It is a well-known problem that the translation of biomedical studies to clinical applications is challenging. A reason frequently discussed in this context is the lack of reproducibility^6,42^. Most notable examples for this include studies about biomarkers^43^ or drug discoveries^44^. The underlying problem is certainly multifaceted but one reason for such problems has been attributed to in vivo animal models^8^.

From a more fundamental point of view, we hypothesize that a cause of the above problems in omics research is related to “emergence”. Put simply, emergence refers to a property of a phenomenon that cannot be explained by the sum of its constituting parts^45^. Formulated differently, the idea of emergence is that “as systems acquire increasingly higher degrees of organizational complexity, they begin to exhibit novel properties that in some sense transcend the properties of their constituent parts, and behave in ways that cannot be predicted on the basis of the laws governing simpler systems”^46^. For biology and medicine, this is of relevance for two reasons. First, both fields are on a higher level of complexity than, e.g., physics and chemistry^47^. Nevertheless, neither field can be explained by the laws of physics. Second, biology and medicine connect a microscopic world with a macroscopic world in the form of a genotype-to-phenotype (GP) mapping^48,49^. Hence, while it is unquestionable that genes and cells are fundamental units of biology, animals and humans express their phenotype on a macroscopic level that defies a straightforward connection between both worlds. The reasons for these problems are generally attributed to the lack of reductionism of biology^50^ and the multi-scale nature of the genotype-to-phenotype mapping^51^. Both problems give rise to emergence.

On a historical note, we would like to remark that Fisher made the simplifying assumption that “genetic inheritance is mainly additive and that all other genetic and environmental contributions to trait variation are deviations from this”^52^. Interestingly, the assumption of additivity is in conflict with the meaning of emergence. This seems to lead to a contradicting situation because of the success of Fisher’s work and the continued usage of similar assumptions, e.g., in modern genome-wide association studies (GWAS)^53^. However, this contradiction is resolved when one distinguishes Mendelian phenotypes from complex phenotypes^54,55^. While Mendelian phenotypes can be successfully studied based on Fisher’s simplifying assumption, as exemplified, e.g., by Cystic fibrosis or Huntington’s disease, complex phenotypes like diabetes, cancer or schizophrenia are different.

More abstractly, we can summarize the above discussion by the following two hypotheses.

**Hypothesis H1** (Mendelian phenotype): A few genes are important in explaining a phenotype.

**Hypothesis H2** (Complex phenotype): All interactions between all genes and all environmental conditions explain a phenotype.

We would like to remark that in omics (studies) usually no explicit formulation of such hypotheses is given. Instead, the tested hypothesis is buried in the conducted study. An immediate consequence of this implicit nature of the underlying hypothesis seems confusions between both which results in the erroneous usage of hypothesis H1 for studies of complex phenotypes. Examples of such studies are omnipresent, e.g., refs. ^56–59^.

We think that a possible reason for the confusion between H1 and H2 is in the misinterpretation of the difference between “a few genes” and “all genes”, and “all interactions”. This difference is crucial because the former cannot be used to study emergent phenomena defying a reductionistic approach, as discussed above. Hence, the problem of contemporary omics studies aiming to investigate a complex phenotype is that they study this based on hypothesis H1 that means reductionistically.

### Appearance of networks

By looking at this problem from a different angle we can obtain a network perspective. Specifically, suppose a study about a complex phenotype found that three genes are playing an important role. Due to the fact that these genes are part of integrated molecular networks they have interaction partners in the form of other genes, respectively, proteins or metabolites. Let’s assume that each of the initial three genes interacts with only five other genes than this results already in a network consisting up to 18 = (3 × 5 + 3) genes. Given that also those genes are part of regulatory networks, each of those genes interacts with further genes. Assuming again five interactions per gene this results in up to 93 = (15 × 5 + 18) interacting genes. This simple example demonstrates that by considering only a few such steps, the resulting network can contain hundreds of genes and by extending this even further than the resulting network will span all active genes in a cell.

This behavior has been observed experimentally. For instance, for the protein-interaction network of human, it has been shown in ref. ^60^ that the average shortest path length is four and in ref. ^61^ the diameter, which corresponds to the largest shortest path length between two nodes, has been found to be 11. Hence, even when starting from only one gene this gene pulls out a network containing all active genes of a cell type. Interestingly, these results consider only the protein-interaction network and not its integration with, e.g., the transcriptional regulatory network and the metabolic network. Hence, one can expect the actual interaction paths to be even shorter.

Another important example is given by studies that focus only on one key gene. Similar to the arguments above, also this gene interacts with a few other genes because otherwise, it could not contribute to the functioning of a cell. In the most extreme case, this gene would interact with only one other gene. However, why would it be justified to emphasize only one of these two genes when reporting results?

The reason for this seems to be historically motivated. Specifically, for early studies of genetics, as conducted, e.g., by Fisher, evolution was of central importance. However, for such studies, the information stored in the DNA is of crucial importance because only this information is directly inherited. In such a context, it makes sense to emphasize the mutation in a gene. Hence, on the DNA level, mutations can be used to single-out individual genes in a sensible way. However, when studying active genes, which translate into proteins or noncoding RNSs, this is no longer possible. The reason for this is that for any type of molecular network, e.g., protein-interaction network, transcriptional regulatory network, gene regulatory network, signaling network or metabolic network^62–64^, at least two entities are needed to form an interaction. Hence, in any type of molecular network, single genes cannot be emphasized without mentioning its interaction partners because without those there would be no interaction and, hence, no contribution of a gene to the functioning of a cell.

In summary, this discussion demonstrates that it is only justified to emphasize individual genes of the DNA level while transcribed or translated gene products form interactions with other gene products and are part of various networks.

### Minimal corroboration vs severe testing in omics

A consequence of the above discussion in the larger context of scientific discovery is that many omics studies do only provide an incremental corroboration for the underlying hypothesis while severe testing occurs rarely^65^. As a reason, the academic incentive structure has been identified favoring the publication of positive results which “propagates an advocacy mindset that is in opposition to the fundamental role of skepticism in science”^66^.

In our opinion, another important reason for this is the confusion between the two hypothesis discussed above, i.e., H1 and H2, which is due to a lack of clarity of Mendelian and complex phenotypes, active and inactive genes, and molecular networks. Generally, those causes are summarized by the term “emergence” which unfortunately seems more of a clouding than enlightenment for the broader community.

Given these problems, it is worth highlighting that there are also examples of severe testing in omics studies. Specifically, the study in ref. ^67^ investigated prognostic gene expression signatures of breast cancer. In order to scrutinize the importance of proposed prognostic biomarkers (PB) the study investigated 48 published signatures, corresponding to established biomarker sets, by generating random gene sets to form new signature sets, as in ref. ^68^. Importantly, these random gene sets were drawn from a gene pool that did neither contain the original signature genes nor any gene involved in the same biological processes as the signature genes, nor proliferation genes. Hence, any random gene set was guaranteed to have no biological similarity to the genes in a signature *S*. By means of survival analysis, it was shown that many random gene sets can be found that have the same prognostic prediction capabilities as the 48 published signatures.

The hypothesis tested by this study can be formulated in the following way:

**Hypothesis PB** (Prognostic biomarkers): A (published) gene expression signature *S* of prognostic biomarkers is important for the biological understanding of breast cancer progression.

In hypothesis PB, biological understanding refers to the collective interactions among all molecular and cellular entities, including mRNAs and proteins and their resulting molecular networks, e.g., protein-interaction network, transcriptional regulation network and gene regulatory network. The key strategy of the above testing is to utilize the (symmetric) association between biological importance and predictability that is generally assumed in biomarker studies^69–71^. Specifically, for prognostic biomarkers that means it is assumed when a signature *S* of prognostic biomarkers is important for the biological understanding, e.g., of breast cancer, then they also have prediction capabilities for the patient's prognosis. Conversely, if one finds from a computational analysis a signature *S* with prediction capabilities for patient prognosis then one concludes that this signature is important for the biological understanding. By substituting a (dedicated) signature *S* with random genes sets, which have per construction no biological similarity with the genes in *S*, hypothesis PB could be falsified for the studied signatures.

We would like to emphasize that above test is severe because the hypothesis is based on published gene expression signatures and the generally assumed association between biological importance and predictability which passed already several other tests which were sufficient to justify the publication of the study.

In the following discussion, we will capitalize on these finding when presenting severe testing for general omics studies.

## A general approach to severe testing

Given the discussion above, we can now formulate a severe testing framework (STF) for omics. The framework consists of three steps. (I) Identification of suitable studies, (II) Identification of a gene pool for severe testing, and (III) Severe testing.

### Identification of suitable studies

**Fig. 3:**
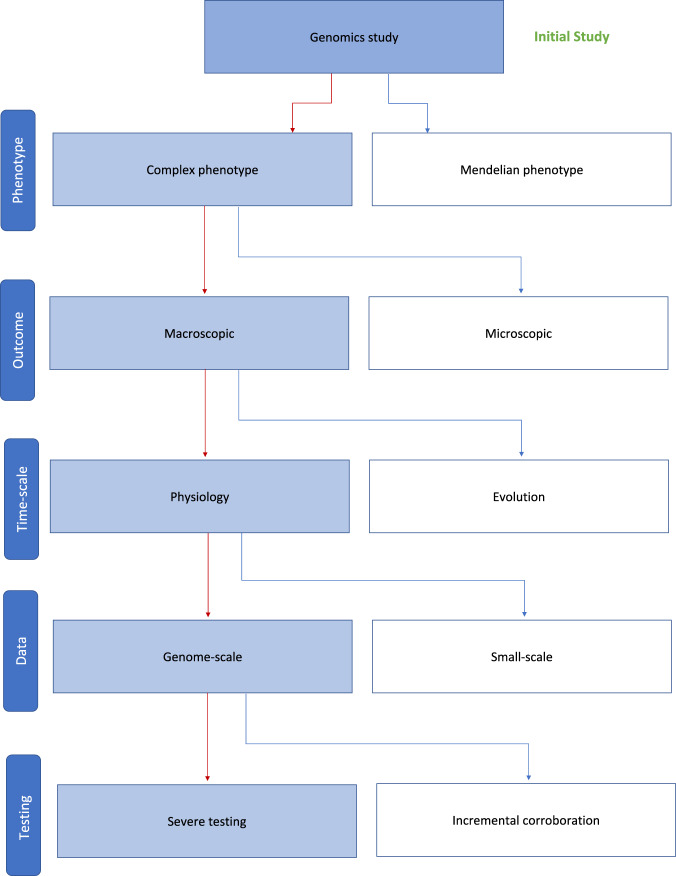
Classification of omics studies to identify prime candidates for severe testing. Each layer performs a decision based on the criterion shown on the left (phenotype, outcome, time scale, data, and testing).

In order to decide if a study is a candidate for severe testing, we provide in Fig. 3 a checklist with five layers. Each of these layers makes a decision if a study may benefit from severe testing, according to our discussion above, or not. We would like to emphasize that the path through this diagram discussed in the following identifies only studies that are prime candidates for severe testing. However, this does not ultimately exclude others.

The first layer is the phenotype distinguishing studies about complex from Mendelian phenotypes. If the phenotype of the study is (likely to be) complex instead of Mendelian, it is a candidate for severe testing. The second layer uses the outcome for a decision. For instance, overall survival, mortality or changes in symptoms indicate a macroscopic level, e.g., for clinical studies, whereas gene expression, protein binding, or mutations point to a microscopic level of an organism. The third layer looks at the timescale of a problem. Here, we distinguish short from long durations of processes, whereas the latter means on an evolutionary scale spanning of many (millions) of generations of an organism. This layer is related to the previous one because for studying the inheritance of genes mutations are playing a central role. The fourth layer checks the available data. This is the only layer effected by the experimental design of a study which is modifiable by planning. For severe testing, genome-scale data are required because only such data allow *possibly* to compensate the activity of some mRNAs/proteins/metabolites by others. Finally, the fifth layer decides about the testing type. While all studies coming from the left side (following the red path) are prime candidates for severe testing it is nevertheless possible to decide against it to perform an incremental corroboration.

As a result from these successive classifications, one obtains studies that are prime candidates for severe testing. We would like to emphasize that this does not ultimately exclude other studies but the justification in favor of severe testing would need to be expanded compared to our arguments. For instance, in order to justify severe testing for a study about a microscopic outcome, shown on layer two in Fig. 3, e.g., about gene expression values, one needs to replace our argument about emergence. Obviously, a study focusing only on a microscopic outcome does not suffer from the problems encountered when bridging from the microscopic to the macroscopic world corresponding to the genotype-to-phenotype mapping (see discussion above), and neither can it make statements about it. If such an argument can be given remains an open question. Since in this paper, our focus is on studies that include an emergent behavior additional side branches in Fig. 3 are not central to our discussion.

### Identification of a gene pool for severe testing

After having identified if a study is a candidate for severe testing, we need to identify which genes to use.

**Fig. 4:**
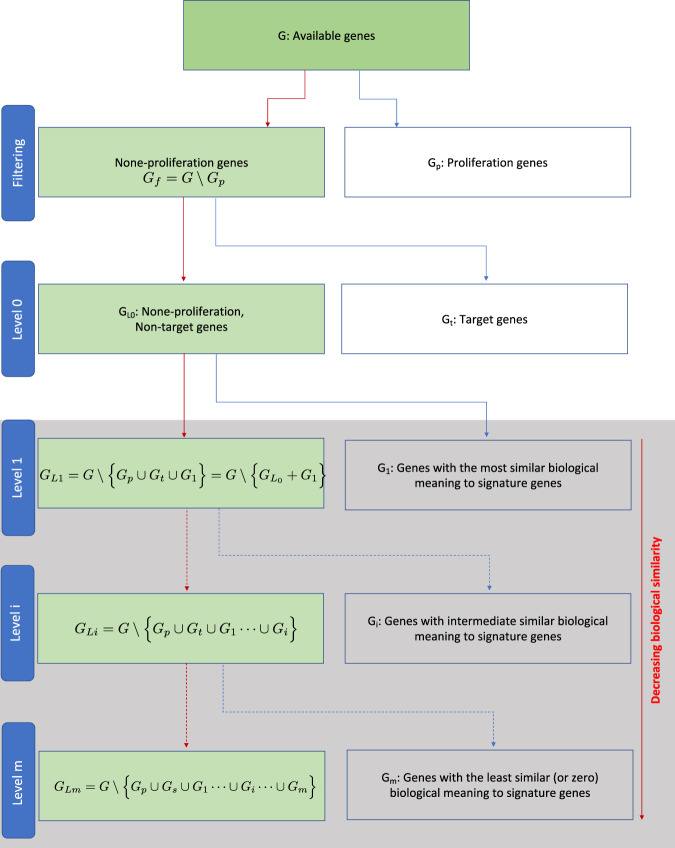
Procedure for preparation of a gene pool for severe testing. Removal of proliferation genes, *G*_*p*_, can be seen as filtering. The genes resulting from each step thereafter (level 0 to *m*) can be used for severe testing, whereas the stringency increases with increasing levels, i.e., the genes on level 1 result in the least stringent test, whereas the genes on level *m* allow the most stringent test.

In order to identify such candidate genes for severe testing, we need to construct a gene pool (visualized in Fig. 4). Let’s denote the set of all available genes by *G*. Here, available genes do not mean all genes that exist for an organism but all genes for which information is available in our data (see layer four in Fig. 3). For these genes *G*, we perform filtering by removing all proliferation genes (indicated by set *G*_*p*_). A reason for this is that it is well-known that the source of variation provided by proliferation genes can lead to a distortion of inference when trying to untangling biological factors affecting cell behavior, e.g., for cell identification^72^ or outcome prediction^73^.

The next step removes all target genes. We indicate the target genes by the set *G*_*t*_. For a study about biomarkers, this may be signature genes (e.g., prognostic, diagnostic, or predictive) or more generally any set of genes that appears of special interest. Due to this characterization, the set of target genes *G*_*t*_ is usually very small, i.e., *G*_*t*_ ≪ *G*. Typical set sizes of *G*_*t*_ range from merely one gene to a few hundred. This leaves us with gene set ${G}_{{L}_{0}}$ containing only genes that are non-proliferation and non-target genes corresponding to ${G}_{{L}_{0}}=G\setminus \left\{{G}_{p}\cup {G}_{t}\right\}$.

Finally, we can remove further gene sets, indicated by *G*_*i*_, according to their biological similarity to the target genes in *G*_*t*_. In general, there are many ways to define biological similarity between genes or sets of genes^74–76^. For instance, in ref. ^77^ a method is provided for obtaining genes associated with gene ontology (GO) levels. This allows a hierarchical exploration of genes that share common GO-terms with the target genes. Regardless what measure is used, successive removal of such gene sets allows to decrease the biological similarity between the remaining genes, given by ${G}_{{L}_{i}}=G\setminus \left\{{G}_{p}\cup {G}_{t}\cup {G}_{1}\cdots \cup {G}_{i}\right\}$, and the target genes *G*_*t*_. For instance, in ref. ^67^ the final level contained only genes in ${G}_{{L}_{m}}$ that had a vanishing biological similarity with *G*_*t*_ corresponding to no common GO-terms. Overall, the above procedure (Fig. 4) allows to construct a gene pool with desired properties which can then be used for severe testing.

### Severe testing

**Fig. 5:**
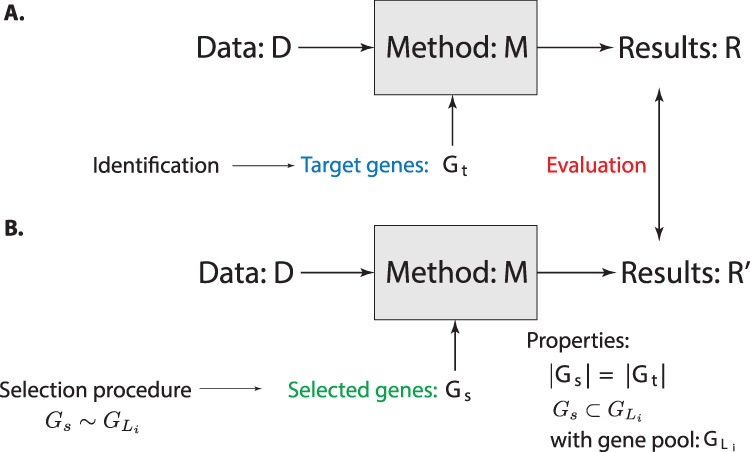
Severe testing for selected genes. **A** Dependency of analysis results (R) on target genes *G*_*t*_. **B** Replication of the same analysis (with the same method *M* and data *D*) by using selected genes, *G*_*s*_, from ${G}_{{L}_{i}}$. The results of both (*R* and $R^{\prime}$) are compared for evaluating the effect of *G*_*s*_.

In Fig. 5, we show the severe testing procedure, whereas Fig. 5A shows the main components of a general analysis. Specifically, a method (M) is applied to a data set (D) leading to results (*R*). The method shall depend on the target genes, *G*_*t*_, by using these as features, e.g., for a classification. In Fig. 5B, we show the same analysis pipeline, however, using now so-called *selected genes*, *G*_*s*_. The selected gene set *G*_*s*_ has two properties. First, its size is the same as of the target genes, i.e., ∣*G*_*s*_∣ = ∣*G*_*t*_∣. Second, *G*_*s*_ is a subset of the gene pool ${G}_{{L}_{i}}$ identified in the previous section, i.e., ${G}_{s}\subset {G}_{{L}_{i}}$. Here *L*_*i*_ corresponds to the level that has been found appropriate.

Now we can formulate the hypothesis of severe testing (ST):

**Hypothesis ST** (severe testing): The results of R (using *G*_*t*_) and the results of R’ (using *G*_*s*_) are not the same.

The implication of hypothesis ST is that if we need to reject it then the genes in *G*_*t*_ and *G*_*s*_ perform indistinguishably. However, due to the different biological meaning of *G*_*t*_ and *G*_*s*_ (see the discussion in the previous section) the biological explanation of the target genes for the obtained results is no longer valid. In case hypothesis ST is rejected, we call the genes in *G*_*s*_ surrogate genes because they provide surrogates for the prediction.

For the above discussion, we assumed that the surrogate genes, *G*_*s*_, are already given. However, how do we obtain them if this is not the case? In general, the selection of the genes in *G*_*s*_ can be seen as a feature selection or optimization problem, and its implementation is problem-specific.

Another problem-specific part of the above STF is the identification of the target genes (see Fig. 5A). However, this is part of the original study we want to scrutinize and for this reason this information is available. Still, for completeness, we would like to mention that, usually, the target genes are found via a method, e.g., for identifying differentially expressed genes^78,79^ or hub genes in a regulatory network^80,81^. However, also biological insights can be used which do not have to be strictly based on formal methods.

## Case studies

In order to demonstrate the validity of the proposed STF, we discuss in the following two examples.

The first study investigated the prognostic gene expression signatures of breast cancer^67^. Specifically, 48 published signatures corresponding to established biomarker sets from the literature were studied by applying the SFT. For this, the random gene sets were drawn from a gene pool according to the procedure in Fig. 4. The gene pool did neither contain the original signature genes nor any gene involved in the same biological processes as the signature genes, nor proliferation genes. Hence, any random gene set was guaranteed to have no biological similarity to the genes in a target signature, *G*_*t*_, as measured by the overlap in GO-terms^82^. Application of survival analysis showed that for each published, established biomarker set many surrogate gene sets can be found that have the same prognostic prediction capabilities. Hence, hypothesis ST needs to be rejected. This demonstrated that none of the 48 studied signatures had a sensible biological interpretation. Furthermore, it is interesting to note that for each established biomarker set not a few but a very large number of surrogate signatures could be found that have the same prognostic prediction capabilities indicating a high redundancy in breast cancer cells. Specifically, it has been shown that this number is in the order of 10^143^ gene sets when making strict assumptions (removing all genes with a GO-term overlap with *G*_*t*_ and proliferation genes) and 10^243^ in the lenient case (remove only the signature and proliferation genes).

The second study investigated the prognostic gene expression signatures of prostate cancer^83^. This study used 32 published prognostic signatures of prostate cancer which were scrutinized following a similar approach as in ref. ^67^ applying the SFT. Also, this study demonstrated that none of the 32 published signatures had a sensible biological meaning. Overall, both studies showed that all 80 studied prognostic signatures serve only as black-box models allowing sensible predictions of prostate cancer outcomes but are not capable of providing causal explanations to enhance the molecular biological understanding of breast and prostate cancer.

Regarding the identification of the genes in *G*_*s*_ it is interesting to note that both studies^67,83^ used a simple selection procedure that performed merely a random selection from the gene pool ${G}_{{L}_{i}}$. While not every random selection resulted in surrogate genes, this procedure was sufficient to find (many) surrogate gene sets *G*_*s*_ as mentioned above. However, other problems may be different, and for this reason, the selection procedure needs to be studied case-by-case.

## The generality of severe testing

We would like to emphasize that the STF discussed in this paper is neither limited to prognostic biomarkers nor to cancer. Instead, the two case studies^67,83^ discussed above should only be seen as instances for its applicability. Importantly, the STF can be utilized for high-dimensional omics studies centered around a few target genes. Typically, such studies involve biomarkers which can be prognostic, diagnostic, predictive, risk, pharmacodynamic/response, safety or monitoring^84^. Examples of high-dimensional omics data other than transcriptomics data are genomics, proteomics, and metabolomics. Hence, any combination of such biomarkers with any type of high-dimensional omics data provide suitable cases amenable for the STF.

Furthermore, any complex disease that cannot be explained by a Mendelian phenotype could benefit from severe testing. Aside from the many different cancer types, those are disorders like Alzheimer’s, asthma, autoimmune, diabetes, multiple sclerosis, Parkinson’s or schizophrenia. Overall, the combinations one can form from (I) different types of biomarkers, (II) different types of high-dimensional omics data, and (III) different complex diseases are enormous, underlining the relevance of the proposed framework.

## Discussion

The above-defined severe testing framework for omics has a few key characteristics which are important to highlight. In the following, we provide a brief discussion thereof.

- **Severe testing is a computational framework:** It is important to note that the introduced severe testing framework is purely computational. That means no additional experiments have to be conducted which would be expensive and time-consuming. Instead, severe testing is based on the data already generated.
- **Severe testing does not require additional methods:** Severe testing uses the same analysis method(s) as the underlying study in utilizing the target genes *G*_*t*_. Schematically, this is highlighted in Fig. 5 where one can see that the same method (M) is used for both cases, just the target genes *G*_*t*_ are substituted by the surrogate genes *G*_*s*_.
- **Severe testing is a natural framework:** It is unquestionable (assuming ethical standards) that all studies strive for faithful results. Hence, any test that helps reaching this goal is supported. Put differently, if one would know a test that would falsify a result, there is not only no reason of not performing this test but it would even violate ethical standards. In this sense, severe testing provides a natural framework for putting results in omics to a test.
- **Severe testing is a practical framework:** When discussing general approaches for scientific discovery, we have seen that the different models are quite intricate theoretically. We have also seen that none of those provides practical approaches but rather general theoretical considerations. In contrast, severe testing provides a practical framework that is directly applicable to studies in omics based on high-dimensional data. Specifically, it provides answers to the questions “what to test” (hypothesis H1 vs H2) and “how to test” (incremental vs severe testing) by a (practical) representation and (a computational) implementation of the abstract concept of falsifiability.
- **Severe testing is constructive:** At first, this may be surprising because falsification is the counterpart of verification and as such usually perceived negatively. However, in contemporary omics we are facing a different situation. Instead of starting from nothing were a falsification could be seen as destroying everything, we start based on the results obtained in the last almost three decades. Specifically, from a Pubmed search one finds over a million published articles about omics, i.e., genomics, transcriptomics, proteomics, and metabolomics; many of which are candidates for the STF. Hence, in omics, severe testing can be seen as topiary by trimming wild-grown hedges to sculptures.
- **Severe testing is different to meta-analysis:** The STF is considerably different to a meta-analysis because the STF does not combine a number of previously obtained results. Instead, it scrutinizes such results individually. Another difference is that in a meta-analysis, a *level of evidence* would be considered, e.g., via *P* values from hypothesis tests. Instead, for the STF the level of evidence needed is an absolute—not relative—one. This means for the STF it is sufficient if, e.g., a set of biomarkers has been identified by a previous study as significant.
- **Severe testing is not an exclusive approach to scientific discovery:** This point is related to the previous one highlighting a different perspective. The STF does not aim to replace, e.g., the hypothetico-deductive method, instead, it complements it. That means the STF does not deal with the process of creating results, which is one part of scientific discovery, but with testing. Hence, it builds on methods of the first part of scientific discovery without restricting them in any way.
- **Severe testing can alleviate reproducibility problems:** Above, we discussed problems with reproducibility in general omics studies and especially in translational research. Application of the STF can help in avoiding such problems because the testing aims at falsifying results and not at confirming. Hence, problems could be identified early, e.g., before clinical trials are performed or animal models are used. That means in order to be efficiently used, the STF should be placed right at the beginning of, e.g., a drug development pipeline after target genes have been identified to avoid problems further downstream.
  However, as a warning, we would like to note that the STF cannot avoid all reproducibility problems. For instance, the STF assumes the availability of published gene signatures which is unfortunately not always the case. Hence, in such a situation, the STF cannot be applied. We would also like to highlight that in the reproducibility problems of studies are multi-facetted, and the STF provides one additional factor to safeguard against it. Hence, STF is not meant to be utilized in isolation but in combination with other measures.

Regarding the last point, we would like to add that in our opinion the replication crisis^85^ is also a lack of the falsification of a hypothesis at an early stage of an investigation.

A final point, we want to highlight relates to a property of the target gene set *G*_*t*_. Above, we mentioned that this set should be small compared to all genes available in a omics data set, i.e., ∣*G*_*t*_∣ ≪ ∣*G*∣. The implication from this is that the available omics data need to be high-dimensional. This high dimensionality is necessary to have a large search space available for the selection procedure to potentially find a proper surrogate gene set *G*_*s*_. Furthermore, it is interesting to note that the absolute size of the target gene set, ∣*G*_*t*_∣, is a coarse indicator if the underlying study was aiming for a Mendelian or non-Mendelian explanation of a phenotype because the extreme boundaries correspond to just one gene (i.e., ∣*G*_*t*_∣ = 1) and all genes (i.e., ∣*G*_*t*_∣ = ∣*G*∣).

## Conclusions

In this paper, we discussed general problems with omics studies for complex phenotypes, including translational research. While these problems are certainly multifactorial, we identified three key factors; one relates to the nature of the problem, and two to the nature of the method of scientific discovery. Specifically, each question about a complex phenotype, defining a specific problem, faces a genotype-to-phenotype mapping which is accompanied by the challenges of emergence and interconnected molecular networks. Furthermore, the two problems of “what to test” (hypothesis H1 vs H2) and “how to test” (incremental vs severe testing) are related to the method of scientific discovery.

For the last three decades, these three issues have been largely ignored by the omics community favoring the generation of many new results of which in retrospect many turned out to be false. In the literature, this has been commonly summarized under the term reproducibility crisis. In order to counteract such problems, we introduced a severe testing framework (STF) that allows to put high-throughput studies centered around a few target genes to be scrutiny. The severe testing framework provides a (practical) representation and (a computational) implementation of the abstract concept of falsifiability and utilizes it in a constructive manner. Particular areas that could benefit from the application of the STF are related to biomarker studies and drug development.
